# Is Resilience Protective of Movement-Evoked Pain in Older Black Women Who Experience Discrimination?

**DOI:** 10.1093/geroni/igab046.2388

**Published:** 2021-12-17

**Authors:** Mayra Astencio, Emily Bartley, Staja Booker, Kimberly Sibille, Josue Cardoso, Adriana Addison, Roger Fillingim, Ellen Terry

**Affiliations:** 1 University of Florida College of Nursing, Miami, Florida, United States; 2 University of Florida, Gainesville, Florida, United States; 5 University of Florida, Pain Research and Intervention Center of Excellence (PRICE), Gainesville, Florida, United States; 6 University of Alabama at Birmingham, BIRMINGHAM, Alabama, United States; 8 University of Florida College of Nursing, Gainesville, Florida, United States

## Abstract

Older non-Hispanic black (NHB) individuals experience greater pain and more frequent experiences of perceived discrimination compared to non-Hispanic white individuals with knee osteoarthritis. The current study explored whether being resilient buffers against movement-evoked pain (MEP) in NHB women who report everyday experiences of discrimination. In a secondary analysis of the Understanding Pain and Limitations in Osteoarthritic Disease (UPLOAD-2) study, data were collected at the University of Florida and the University of Alabama at Birmingham. Participants were 58 community-dwelling older women who self-identified as NHB and reported knee osteoarthritis. Participants completed the Brief Resilience Scale, a self-report measure of trait resilience. MEP was assessed following the Short Physical Performance Battery. Moderation analyses were conducted to investigate whether resilience moderates the association between experiences of discrimination and MEP. Study site, age, body mass index, and income were included as covariates. Overall, neither everyday experiences of discrimination (b=.292, 95% confidence interval [CI]=-.415 to 1.000) nor trait resilience was associated with MEP (b=-11.540, 95% CI=-23.583 to .503). However, there was a significant interaction (b=1.037, 95% CI=.150 to 1.925) between experiences of discrimination and trait resilience in predicting MEP. Simple slopes analysis revealed that lower discrimination was associated with lower MEP, but only in women who reported high levels of resilience (b=1.100, p=.014), but this protective effect of resilience was absent in women reporting high discrimination. Our findings suggest that as discrimination increases, the protective effects of resilience on movement evoked-pain decreases. Therefore, high trait resilience may be protective when experiences of discrimination are low.

